# Successful Use of Tofacitinib in Refractory Takayasu Arteritis: A Case Series

**DOI:** 10.31138/mjr.230929.su

**Published:** 2023-07-31

**Authors:** Prakashini MV, Debashis Maikap, Prasanta Padhan

**Affiliations:** Department of Clinical Immunology and Rheumatology, Kalinga Institute of Medical Sciences, KIIT University, Bhubaneswar, Odisha, India

**Keywords:** Takayasu, large vessel vasculitis, tofacitinib, glucocorticoids, angiogram

## Abstract

**Objective:**

To study the clinical effectiveness and safety of Tofacitinib in refractory Takayasu arteritis (TAK).

**Methods:**

This study was conducted from September 2021 to June 2022. Ten cases of refractory TAK patients were enrolled. TAK patients who required >7.5mg prednisolone or equivalent per day and those who failed to achieve remission despite being on conventional immunomodulators, with an Indian Takayasu Activity Score 2010 (ITAS 2010) of > 1 were included in this study. Tofacitinib was used at a dose of 5 mg twice daily after ruling out latent tuberculosis. The patients were followed up at 1, 3 and 6 months. ESR, CRP and ITAS 2010 were recorded at each visit. Complete blood counts, liver, and kidney function tests were done to assess the adverse effects at baseline and follow up.

**Results:**

There was a mean decline in ESR from 60.7 ± 20.05 mm/1^st^ hour at baseline to 11.9 ± 2.38mm/1^st^ hour at 6 months, CRP from 28.9 ± 16.77 mg/L at baseline to 6.8 ± 7.52 mg/L at 6 months, ITAS 2010 from 6.2 ± 2.74 at baseline to 0.6 ± 1.26 at 6 months (p value 0.016). All the patients tolerated tofacitinib well without any adverse effects.

**Conclusions:**

The results of our research indicate that tofacitinib is safe and effective for treating patients with refractory TAK.

## INTRODUCTION

Takayasu arteritis (TAK), otherwise called pulseless disease or Martorell syndrome, is a systemic granulomatous large vessel vasculitis mainly affecting young women (<40 years) in the Asian countries. TAK presents with varied manifestations, ranging from constitutional symptoms to vascular symptoms like unequal arm blood pressure measurements, vascular bruit, and ischemic complications like stroke. Vascular constriction and progressive intimal fibrosis associated with chronic inflammation of TAK mostly affect the aorta and its branches, coronary, and pulmonary arteries.^1^ Insufficient perfusion (or even ischemia) of the associated tissue or organ might result from the involved arteries being stenotic or blocked. This inflammatory process is hypothesised to be a result of aberrant activation of the immune system, with both innate and adaptive immune mediators contributing to the pathogenic process.^1^ Genetic influence in the pathogenesis has been described, with HLA-B*52 allele being at the crux of it, with its prevalence being high among Asians, particularly the Japanese, where TAK is common (40 cases per one million population); HLA-B*52 allele is detected in as many as 10% of the population in Japan.^3^ Owing to the scarcity of randomized placebo-controlled trials in TAK, the treatment has by far relied on evidence from case series, open studies, and expert opinions. The cornerstone of therapy and for remission induction are glucocorticoids. The use of methotrexate, leflunomide, mycophenolate mofetil, azathioprine, and cyclophosphamide in TAK^4–6^ is supported by evidence from uncontrolled prospective and retrospective case series, and between 60 and 80 percent of patients can achieve clinical remission.^7^ However, the significant rate of relapse (>50%) and disease progression seen on imaging, point to the need for more efficient therapies.^8^ Despite the lack of good quality studies supporting the use of disease modifying anti-rheumatic drugs (DMARDs), it is advised to start adjunctive conventional immunosuppressives at the time of diagnosis.^9^ Only two randomized controlled trials for the use of bDMARDs in TAK have been conducted to date, where abatacept (CTLA-4 antagonist) failed to show significant time in reduction to relapses^10^; however, tocilizumab was promising as it showed a trend towards reduction in the time to relapse.^11^ The superiority of biologicals over traditional DMARDs in TAK, however, has not yet been supported by high-quality evidence. The choice of second-line drugs includes TNF inhibitors or tocilizumab in patients with relapses.^9^ Tofacitinib is a JAK 1/3 inhibitor, which recently has been demonstrated to be effective in large vessel vasculitis in animal models, suppressing tissue-resident T-memory cells and preventing microvascular angiogenesis.^12^ JAK/STAT signalling is essential in TAK vascular fibrosis mediated by IL-6.^13^ Additionally, it has been demonstrated that JAK inhibitors increase T-regulatory cells in TAK patients while decreasing subsets of T helper (Th) 1 and Th17 cells.^13^ One observational study, one prospective study and two case reports have highlighted the use of tofacitinib leading to reduction or prevention of relapse, along with tapering of glucocorticoid dosage to < 7.5mg/day of prednisolone or equivalent in TAK treatment.^14–17^ Hereby we report 8 out of 10 cases of refractory TAK who were successfully treated with tofacitinib.

## OBJECTIVES

The aim of this study was to find out the effectiveness and safety of Tofacitinib in refractory Takayasu arteritis (TAK).

## MATERIALS AND METHODS

This study examined the effectiveness and safety of tofacitinib 5 mg twice daily in 10 patients with refractory TAK from September 2021 to June 2022. All patients having a verified diagnosis in accordance with the 1990 American College of Rheumatology’s modified classification criteria^18^ and the Chapel Hill Consensus Conference^19^ with an Indian Takayasu Activity Score 2010 (ITAS 2010) of >1^20^ were included. ITAS 2010 is an accepted scoring system for the assessment of disease activity in TAK, and has been validated for inter-rater reliability, convergence with Birmingham Vasculitis Activity Score (BVAS), correlation with Physician’s global assessment (PGA) and ESR/CRP.^20^ It is sensitive to change and is a reliable measure of disease activity for monitoring of the patients. Higher ITAS 2010 scores suggest poor control of active disease by current therapy (**Supplementary data Figure 1**).

At baseline, all patients were receiving glucocorticoids (GCs) at a dose of >7.5 mg prednisolone or equivalent per day and had failed to respond to conventional immunomodulators for more than 6 months. They had received one or more of the following therapies – 20–25mg of Methotrexate weekly or 100–125mg/day of Azathioprine or 2g/day of Mycophenolate mofetil (**Supplementary data Table 1**). Latent tuberculosis was ruled out prior to starting tofacitinib by plain chest radiograph, Interferon Gamma Release Assay (IGRA) and tuberculin skin test (TST).

**Table 1.** Clinical manifestations of patients with TAK receiving Tofacitinib at baseline and during follow up. None of the patients had occlusion of the vessel lumen.

**Patient number**	**Age (years)**	**Sex**	**Disease duration (months)**	**Symptoms /sign suggestive of active disease**	**Vessel lesions (by MR angiogram)**		**Numano angiographic classification**	**Immuno-suppressant drug before Tofacitinib**	**Glucocorticoid dose before starting Tofacitinib (prednisolone or equivalent, mg/day)**	**Parameter assessed**	**Baseline**	**1 month**	**3 months**	**6 months**	**Glucocorticoid dose at 6months of tofacitinib (prednisolone or equivalent, mg/day)**
					Stenosis	Thickened vessels									
1	15	F	12	Headache, hypertension, fatigue	Rt-SCA, Lt-SCA	NA	V	MTX, MMF	20	ESR (mm/hr)	64	32	30	12	5
										CRP (mg/l)	16	10	4	3	
										ITAS	5	3	3	0	
2	26	F	36	Headache, fatigue, hypertension, limb claudication	Rt-RA, Lt-RA, Lt-CCA, Abd-AO, CA	Diffuse Th-Ao, Abd-Ao	V	MTX, MMF	15	ESR (mm/hr)	44	24	12	10	15
										CRP (mg/l)	24	18	12	5	
										ITAS	11	7	4	3	
3	15	F	18	Headache, carotidynia, syncope	Lt-CCA, Rt-CCA, Rt-SCA, PA	Arch of Ao, Abd-Ao	IIB	MTX, MMF	20	ESR (mm/hr)	25	20	15	10	10
										CRP (mg/l)	54	28	14	28	
										ITAS	9	4	3	3	
4	27	F	14	Hypertension, headache	Abd-Ao, Rt-RA, Lt-RA	Abd-Ao	IV	MTX, MMF	10	ESR (mm/hr)	66	33	20	15	5
										CRP (mg/l)	11	5	4	4	
										ITAS	4	2	0	0	
5	42	M	40	Hypertension	Ao, Rt-RA, Descending Tho-Ao	Abd-Ao	IV	AZA, MMF, MTX	20	ESR (mm/hr)	54	32	20	12	5
										CRP (mg/l)	33	20	11	6	
										ITAS	4	0	0	0	
6	27	F	26	Headache, dizziness, syncope, seizure, carotidynia, bilateral upper limb and jaw claudication, amaurosis fugax.	Rt-CCA, Lt-CCA, Rt-SCA, Lt-SCA, Ascending-Ao	Rt-SCA, Lt-SCA, Rt-CCA, Lt-CCA	IIB	MTX, MMF	20	ESR (mm/hr)	85	65	20	14	5
										CRP (mg/l)	42	32	25	4	
										ITAS	10	6	2	0	
7	31	F	20	Hypertension	Rt-RA, Lt-RA	NA	IV	MTX	20	ESR (mm/hr)	67	50	20	11	5
										CRP (mg/l)	16	14	10	4	
										ITAS	6	2	1	0	
8	43	F	38	Hypertension	Abd-Ao	Rt-RA, Lt-RA	IV	MTX, MMF	10	ESR (mm/hr)	74	40	37	9	5
										CRP (mg/l)	12	10	6	5	
										ITAS	4	1	0	0	
9	26	F	24	Hypertension, headache	Abd-Ao	NA	III	AZA, MMF, MTX	15	ESR (mm/hr)	40	32	18	16	5
										CRP (mg/l)	25	16	4	3	
										ITAS	4	2	2	0	
10	31	F	24	Visual blurring, carotidynia, mid back pain, headache, fever, jaw claudication	Rt-CCA, Lt-CCA	Abd-Ao	IIB	MMF, Etanercept	20	ESR (mm/hr)	88	28	14	10	5
										CRP (mg/l)	56	11	6	6	
										ITAS	5	2	2	0	

Abd: Abdominal; Ao: Aorta; AZA: Azathioprine; CA: Celiac axis; CCA: Common carotid artery; CRP: C-Reactive Protein; ESR: Erythrocyte Sedimentation Rate; ITAS: Indian Takayasu Activity Score; Lt: Left; MMF: Mycophenolate mofetil; MTX: Methotrexate; RA: Renal Artery; Rt: Right; SCA: Subclavian artery; Tho: Thoracic

The total follow-up duration was 6 months. Clinical manifestations, adverse events, CRP level, erythrocyte sedimentation rate (ESR), and ITAS 2010 were all documented at each visit. All patients’ complete blood counts, renal functions and liver functions were done at baseline, 1 month, 3 months and 6 months. The response to treatment was defined as^21^: (1) complete remission (CR), defined as the absence of disease activity (ITAS 2010 = 0); (2) partial remission (PR), defined as ITAS 2010 = 1; and (3) persistently active disease, defined as inability to attain ITAS 2020 = 1 or persistently raised CRP after 4–6 weeks of treatment. We defined relapse as any new clinical manifestations or increase in ITAS after achieving CR or PR during the study.

## RESULTS

**Figure 1.**
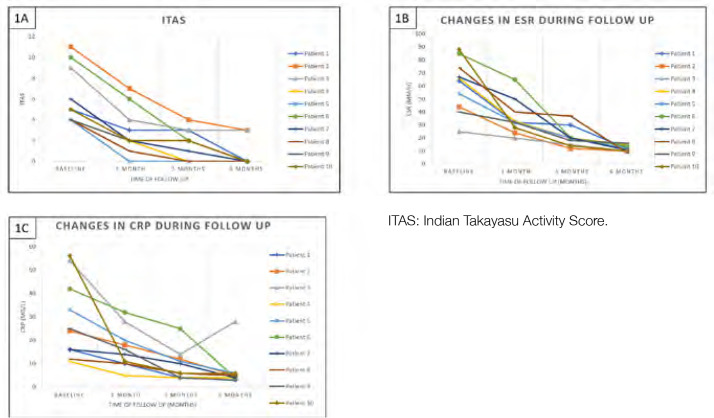
**(A)** Changes in disease activity score (ITAS 2010) during patient follow up. **(B)** Changes in ESR levels during Tofacitinib treatment. **(C)** Changes in CRP levels during Tofacitinib treatment.

**Table 1** shows the demographics and clinical symptoms of the 10 TAK patients. The disease was female predominant with F:M = 9:1. The mean duration of disease was 25.2 ± 9.89 months. According to Numano angiographic classification,^22^ 3 patients had Type II TAK, 1 patient had Type III TAK, 4 patients had Type IV TAK and 2 patients had Type V TAK. The average GC dose before starting tofacitinib was 17.8 ± 3.63 mg of prednisolone or equivalent per day. The mean ITAS 2010 at baseline, 1 month, 3 months and 6 months were 6.2 ± 2.74, 2.9 ± 2.18, 1.7 ± 1.41 and 0.6 ± 1.26 respectively, with the decline in ITAS2010 being statistically significant with a p-value of 0.016 (**Figure 1A**). The mean ESR (mm/1^st^ hour) at baseline, 1 month, 3 months and 6 months of tofacitinib were 60.7 ± 20, 35.6 ± 13.21, 20.6 ± 7.56 and 11.9 ± 2.37 respectively (**Figure 1B**). The mean CRP (mg/L) at baseline, 1 month, 3 months and 6 months of treatment were 28.9 ± 16.76, 16.4 ± 8.43, 9.6 ± 6.53 and 6.8 ± 7.52 respectively (**Figure 1C**). The mean GC dose at 6 months of treatment with tofacitinib was 6.5 ± 3.37 mg of prednisolone or equivalent per day. Eight patients achieved CR (ITAS 2010 = 0), while two patients had a persistently active disease. Mean ITAS 2010 at baseline, 1 month, 3 months and 6 months in the patients who achieved CR was 5.25 ± 1.26, 2.2 ± 1.64, 1.25 ± 1.16 and 0 respectively. Conversely, it was 10 ± 1.14, 5.5 ± 2.12, 3.5 ± 0.7 and 3 in the two patients with persistently active disease. The 2 patients who had persistently active disease, required a higher dose of 12.5 ± 3.53 mg/day, whereas the 8 patients who achieved CR were on a GC dose of 5 mg/day at 6 months. None of our patients relapsed during follow-up. There were no adverse events during therapy.

## DISCUSSION

In our study, 8 out of 10 consecutive patients of refractory TAK responded to tofacitinib. Although refractory TAK has no standardised definition, the Turkish TA Study Group defined refractory disease as “angiographic or clinical progression despite treatment or the presence of any of the following characteristics: (i) prednisolone dose >7.5 mg/day after 6 months of treatment, despite administration of conventional immunosuppressive agents; (ii) new surgery due to persistent disease activity; (iii) frequent attacks (more than three per year) and (iv) death associated with disease activity”.^23^ All our patients were on 10 to 20 mg of prednisolone or equivalent per day and the attempt to taper it to <7.5mg/day was unsuccessful. All of them had evidence of active disease (as described in **Table 1**) when tofacitinib was started. The failure to achieve CR in two patients in our study may be attributed to the higher mean ITAS 2010 at baseline. A higher dose of tofacitinib 10mg twice a day with long-term follow-up of these patients is required to further ascertain the treatment response.

Following treatment with tofacitinib, improvements in ITAS and inflammatory markers were seen in eight patients. Although biologic therapies could have been offered to our patients, cost was a limiting factor for the same. According to Tombetti and Mason,^27^ every six to twelve months for the first two years of TAK treatment, the response is commonly assessed by MR-Angiogram (MR-Ang) or CT-Angiogram. Once clinical remission is sustained, annual MR-Ang monitoring is advised. According to the 2018 EULAR guidelines,^9^ if a flare is suspected, imaging should be performed again; however, in cases of clinical or laboratory remission, repeat imaging is not necessary. As per one study by Goel and Danda et al,^28^ the structural changes in the vessel to observe the treatment response would be evident only after 1 year, which was one of the reasons we did not repeat the angiogram at 6 months owing to high cost and limited availability.

In TAK patients, JAK/STAT-, cytokine/chemokines-related genes and interferons are highly upregulated in CD4+ and CD8+ T cells.^24^ JAK inhibitors have shown promising results in TAK in a few isolated case reports and small case series.^14–17^ Type 1 and 2 interferons, IL-6, IL-12, IL-17, and IL-23, which are the most common cytokines implicated in TAK pathogenesis are suppressed by JAK inhibition.^25^ Additionally, JAK inhibitors, including tofacitinib also target macrophages and natural killer cells which are implicated in the pathogenesis.^26^

During follow-up, none of the patients experienced any adverse effects with tofacitinib. No patient experienced any viral (herpes zoster) or bacterial infections throughout six months, and complete blood counts, renal functions, and liver functions were all normal.

Our study was limited by the small number of patients, single-centre, and a short follow-up duration. The results of our study indicate that tofacitinib is safe, effective and can reduce the need for GCs in patients with refractory TAK. To further support the effectiveness and safety of tofacitinib treatment for individuals with refractory TAK, a prospective randomised controlled clinical trial is necessary.

## CONCLUSION

Targeting the upregulated JAK/STAT pathway with tofacitinib yielded positive results with eight out of ten patients achieving complete remission by 6 months of initiation of treatment. This study highlights that tofacitinib is a safe and effective therapeutic choice in refractory TAK patients, particularly in a resource-constrained nation like India.

## ABBREVIATIONS

CR: Complete remission
CRP: C-reactive protein
CTLA: Cytotoxic T-lymphocyte associated antigen
DMARD: Disease Modifying anti-rheumatic drug
bDMARD: Biological Disease Modifying anti-rheumatic drug
ESR: Erythrocyte sedimentation rate
GC: Glucocorticoids
HLA: Human Leucocyte antigen
IL: Interleukin
IGRA: Interferon gamma release assay
ITAS: Indian Takayasu activity score
JAK/STAT: Janus kinase/Signal transducer and activator of transcription
MR-Ang: Magnetic resonance angiography
PR: Partial remission
TAK: Takayasu arteritis
TST: Tuberculin skin test
TNF: Tumour necrosis factor
